# Neurotensin and Its Involvement in Female Hormone-Sensitive Cancers

**DOI:** 10.3390/ijms252111648

**Published:** 2024-10-30

**Authors:** Ninon Bertrand, Romane Mougel, George Riley, Marie Bruand, Guillaume Gauchotte, Mikaël Agopiantz

**Affiliations:** 1 Department of Gynecology and Obstetrics, CHRU de Nancy, Université de Lorraine, F-54000 Nancy, France; n.bertrand@chru-nancy.fr; 2 Department of Fertility Medicine, CHRU de Nancy, Université de Lorraine, F-54000 Nancy, France; r.mougel@chru-nancy.fr; 3 Department of Endocrinology, Diabetes and Nutrition, CHRU de Nancy, Université de Lorraine, F-54500 Vandœuvre-lès-Nancy, France; g.riley@chru-nancy.fr; 4 Department of Radiation Therapy, Institut de Cancérologie de Lorraine, F-54500 Vandoeuvre-lès-Nancy, France; m.bruand@nancy.unicancer.fr; 5 Department of Pathology, CHRU de Nancy, Université de Lorraine, F-54500 Vandœuvre-lès-Nancy, France; g.gauchotte@chru-nancy.fr; 6 INSERM UMRS 1256, Nutrition, Genetics, and Environmental Risk Exposure (NGERE), Université de Lorraine, F-54500 Vandœuvre-lès-Nancy, France

**Keywords:** neurotensin, breast cancer, ovarian cancer, endometrial cancer

## Abstract

Neurotensin (NT) is a peptide involved in digestion, neuromodulation, and cancer progression. NT and its receptors (NTR1 and SORT1 mainly) have been widely studied in oncology. Data show that NT expression is under the control of sex steroid hormones, in particular estradiol. We focused on its involvement in three main female hormone-sensitive cancers, breast, ovarian, and endometrial cancer, in a narrative review. NT, NTR1, and SORT1 are mostly expressed in these three cancers, and their involvement in oncologic processes such as proliferation and invasion seems to match, as does their impact on prognosis for most. The development of NT receptor-targeted therapies, including theranostics and radioligand treatments, presents a promising avenue for personalized cancer treatment.

## 1. Introduction

Neurotensin (NT) is a 13-amino-acid peptide originally isolated from the bovine hypothalamus by Carraway and Leeman in 1973 [1]. NT is found both within the central nervous system and in the gastrointestinal tract. On one hand, in the brain, NT has shown different roles, such as neuromodulation of dopamine transmission [2] or hypothermia and analgesia [3]. Thus, it has been studied in different pathologies such as schizophrenia [4] and Parkinson’s disease [5]. On the other hand, in the digestive system, it exerts a variety of effects including the inhibition of gastric and small-bowel motility or the facilitation of fatty acid translocation [6]. NT’s role as a growth factor in many tissues has been widely studied, as has its involvement in cancer [7].

These different effects are mediated through three receptors. The first NT receptor (NTR1) was cloned in 1990 by Nakanishi et al., who showed that it belonged to the family of G-protein-coupled receptors [8]. Similarly, NTR2 also belongs to the G-protein-coupled receptors and has a lower affinity for NT [9]. NTR3, or sortilin (SORT1), is structurally unrelated to the first two and is characterized by a single transmembrane domain [10]. In tumors, NTR1 is considered the main receptor presiding over the effects of NT, specifically those on cell proliferation, migration, and invasion [11]. NTR2 shows a localized distribution, and its expression has been detected in prostate cancer [12]. Unlike NTR1 and NTR2, which are NT-specific, NTR3 binds to many other factors and is known to also be involved in tumor progression [13].

NT and its receptors have been widely studied in different neoplastic tissues. Deregulation of the neurotensinergic pathway has been observed in numerous cancers, such as colonic adenocarcinoma [14], small-cell lung cancer [15], medullary thyroid carcinoma [16], hepatocellular carcinoma [17], pancreatic cancer [18], breast cancer [19], and non-small-cell lung cancer [20]. Here, NT is vastly overexpressed compared to corresponding normal tissues. Aberrant overexpression of NTR1 is also detected in the majority of solid cancers and cancer cell lines, including when it is absent or very weakly expressed in corresponding normal tissues, as in breast cancer [19,21], colon cancer [22], pancreatic cancer [18], prostate adenocarcinoma [23], and non-small-cell lung cancer [20]. Our team has previously shown that NTR1 expression is linked to the prognosis of hormone-sensitive endometrial and ovarian adenocarcinomas [24,25].

Additionally, research has been conducted concerning the direct link between sex steroid hormones, especially estrogens, and NT. A high correlation was found between NT and estrogen receptor (ER) α expression [26,27,28]. Moreover, older and more recent data have shown that NT is expressed and upregulated by estradiol, both in the hypothalamus, where it is involved in E2-induced negative feedback mechanisms, and in breast tissue [19,29,30]. Finally, NT involvement in reproductive functions has been clearly demonstrated [31]. NT participates in an autocrine manner in the mechanisms of ovulation via NTR3; spermatozoa express only its receptors, whereas in the female reproductive system both NT and its receptors are expressed, enhancing the acrosome reaction of spermatozoa in mammals [31].

Since breast, ovarian, and endometrial cancers are the most frequent hormone-sensitive female cancers, we wished to review the data concerning theses three cancers and the involvement of NT, allowing us to synthesize what is known concerning the different processes of these cancers, open prognostic and therapeutic perspectives, and research avenues for the future.

## 2. Expression of NT and Its Receptors in Hormone-Sensitive Cancers

### 2.1. Breast Cancer

The expression of the three different receptors has been assessed in different breast cancer human cell lines.

First, Somaï et al. showed NTR1 overexpression in MCF-7 cells (estrogen receptor(ER)-positive cells) via RT-PCR, while it seemed absent in normal epithelial breast cells [32], in accordance with Souazé et al. [21]. NTR1 and NTR2 expressions were confirmed using Western blot in MCF-7 cells. Using indirect immunofluorescence, NTR2 expression was membranous, unlike NTR1 which was intracytoplasmic and granular, associated with NT pathway activation [33]. NTR1 expression was also detected in MDA-MB-231 (triple-negative (TN) cell line) cells using RT-PCR and immunofluorescence assays [34]. Conversely, NTR2 expression was variable across the different breast cancer cell lines; Western blot showed strong NTR2 expression in ER-positive breast cancer cells, while it was almost absent in TN cancer cells [33]. In a series of 70 patients who underwent tumorectomy or mastectomy for invasive ductal carcinoma (IDC) with concomitant ductal carcinoma in situ (DCIS), 91% were positive for NTR1, with a threshold of 10% stained cells. Among those 70 patients, 50 were then studied, and 30% showed co-expression of NT and NTR1 in immunohistochemistry [21]. Similarly, in the invasive component of 106 samples of IDC studied by Dupouy et al., the majority showed a high proportion of NTR1-positive cells (from 50 to 100%) using immunohistochemistry, and even 38 patients (35%) exhibited very high NTR1 expression (≥80% of tumor cells). Additionally, the subpopulation coexpressing NT with high expression of NTR1 was significantly correlated with positive ER expression [19]. Moreover, NT expression in normal epithelial breast cells is regulated by estradiol, as an enhancement of transcripts was observed when those cells were exposed to estradiol, and this effect was abolished when exposed to a pure anti-estrogen [19].

SORT1 expression has been well assessed in TN breast cancer cells using Western blot [35,36]. Nevertheless, it was also assessed in different breast cancer cell lines, including luminal A (MCF-7) and Human Epidermal Growth Factor Receptor-2 (HER2)-positive (SKBR3) cancer cell lines [37]. Similarly, high expressions of SORT1 were assessed in TN breast cancer samples [36]. This finding was confirmed in an immunohistochemistry analysis in a series of 318 clinically annotated breast cancers and 53 adjacent normal tissues, showing that most normal tissues presented low levels of SORT1, while the proportion of cases with intermediate or high levels increased in cancers. Indeed, 66% were positive for SORT1 regardless of ER and progesterone receptor (PR) expression or the different molecular subtypes of breast cancer (luminal A and B, HER2+, TN) [37].

### 2.2. Ovarian Cancer

NT expressions, as well as NTR1 and weak NTR2 expressions, were assessed in human ovarian cancer cell lines [25]. In fact, if NT was expressed, in both SKOV3 and A2780 (epithelial ovarian cancer) cell lines, NTR1 mRNA was only detected in SKOV3 cells. Immunohistochemistry studies were then performed on 46 ovarian cancer samples, assessing NTR1-positive expression for 72% of them and 74% when it came to NT. By contrast, 10 samples from nonmalignant tissues were analyzed and 9 of them showed negative or very weak staining. 

SORT1 expression was assessed in many ovarian carcinoma cell lines, including SKOV3, using Western blotting. This expression was confirmed using RT-PCR and indirect immunofluorescence staining on ovarian carcinoma samples, while it was either absent or significantly lower in healthy tissues when assessed using various techniques [35,38,39].

### 2.3. Endometrial Cancer

NT’s presence was assessed in cows’ [40] and goats’ endometrium samples, where estrogen and progesterone regulate its mRNA levels [41]. NT and NTR1 expression was assessed in human endometrial adenocarcinomas and nonmalignant samples [24]. While 48 benign samples out of 66 were positively labeled with an NTR1 antibody, with weak staining in most of them, NTR1 was significantly overexpressed in cancer samples compared to nonmalignant tissues with global staining nearly 14-fold higher in endometrial adenocarcinomas using immunohistochemistry. Indeed, 90 of the 100 cancer samples were positively labeled with an NTR1 antibody, with global staining significantly increasing with grade. In the same way, all of the cancer samples showed a large amount of cells that were positively labeled with the anti-NT antibody and thus significantly higher expression in endometrial adenocarcinomas than in benign samples [24].

Similarly to breast and ovarian cancer, high SORT1 expression was assessed in endometrial cancer cell lines (HEC-1-A, HEC-1-B, AN3-CA, SK-UT-1B, and KLE cell lines) using Western blot. Increased SORT1 staining was also seen in endometrial tumors compared to normal endometrial tissues, as 10 out of 12 (83%) samples showed higher expression levels of SORT1 than in healthy tissues [35].

In summary, the expression of NT and its receptors has been thoroughly investigated in hormone-sensitive cancers. In breast cancer, NTR1 and SORT1 appear overexpressed in human samples and in human cancer cells regardless of hormone-sensitive lines. Similarly, ovarian cancer studies reveal variable NTR1 expression between different cell lines and SORT1 expression in many cancer cell lines, with significant overexpression in malignant tissues compared to nonmalignant ones concerning both NTR1 and SORT1 expression. Likewise, in endometrial cancer, NT, NTR1, and SORT1 appear overexpressed, with NTR1 levels increasing with tumor grade, while their expression is lower or absent in normal tissues. The main data about NT expression and its receptors are summarized in Table 1.

## 3. Oncologic Processes Involved

The main processes involved in tumor progression via activation of the NT/NTR1 complex are cell proliferation, survival, migration, invasion, and neoangiogenesis. NT induces proliferation stimulation in tumor cells from pancreatic, colon, prostate, and small-cell lung cancer [42,43]. Transcriptome analysis of NT-treated exposed pharyngeal squamous cell carcinoma confirmed the overexpression of genes involved in the metastatic process, such as matrix metalloproteinase (MMP-1) and interleukin IL-8 [44]. Similarly, NT accelerates the migration and invasion of colon [45] and pancreatic [46] cancer cells. Finally, NT can promote neoangiogenesis via the urokinase receptor [47], IL-8, and CXCL1 (CXC Motif Chemokine Ligand 1), with simultaneous activation [48].

Similarly, SORT1 is involved in different oncologic processes, such as proliferation and migration in hepatocellular carcinoma [49] or pancreatic cancer cell invasion [50]. It was even recently shown to exert a role in vasculogenic mimicry, both in ovarian and breast cancer cells [51]. Nonetheless, SORT1 effects are mediated through a variety of different ligands, and if its role in NT-mediated oncologic processes in the colon, prostate, or pancreas has been well documented [52], the data concerning the NT/SORT1 complex in female hormone-sensitive cancer are weak.

### 3.1. Cell Growth and Proliferation

Proliferation is the most studied process in the literature concerning NT and its receptors. It has been well documented in both hormone-sensitive and TN breast cancer. For instance, the synthetic NT analogue JMV-449 increased DNA synthesis in MDA-MB-231 cells [53]. In hormone-sensitive breast cancer cells, JMV-449 also stimulated proliferation by inhibiting apoptosis through Bcl-2 proto-oncogene activation, mediated by the MAPK pathway [32]. In xenografted mice with ER-positive tumors, NT overexpression accelerated tumor growth [54]. The NT/NTR1 complex induces the overexpression of EGFR, HER2, and HER3. This synergic effect is confirmed with tumor growth restrained by Lapatinib, a tyrosine kinase inhibitor targeting the HER2 and EGFR pathways. Similarly, in TN breast cancer, silencing NTR1 in MDA-MB-231 xenografts resulted in reduced tumor volume and increased tumor doubling time [21]. Comparable results were achieved using SR48692, a specific NTR1 antagonist, further confirming NTR1’s role in TN breast tumor growth.

NT also plays a role in ovarian carcinoma proliferation. Exogenous NT treatment increased proliferation in OCAR3, a high-grade serous ovarian carcinoma cell line [55]. SORT1 silencing in Caov-4 cells (another high-grade serous ovarian carcinoma line) led to increased apoptosis and decreased proliferation [39]. Interestingly, a heterodimerization between NTR1 and SORT1, leading to NT internalization, has been described in colonic adenocarcinoma cell lines [56], making NT/NTR1/SORT1 a potential therapeutic target for ovarian carcinoma.

### 3.2. Invasion and Metastatic Potential

NT and its receptors are involved in tumor invasion and metastasis. In breast cancer, Dupouy et al. demonstrated that ER-positive NT-negative MCF-7 xenografts did not develop metastases, while NT-overexpressing clones developed metastases in 41% and 76% of cases for low- and high-NT-expressing clones, respectively [54]. Mechanistically, the NT/NTR1 complex promotes pro-metastatic cellular changes by reducing basal cell adhesion, a critical factor for metastatic spread, and synergizes with EGF to enhance cell migration and invasion. In TN breast cancer, NT stimulates invasion and migration through NTR1 activation, leading to matrix metalloproteinase-9 (MMP-9) expression, which is crucial for invasion [21,53]. NTR3 also plays a role in TN cell invasion, as its knockdown in MDA-MB-231 cells inhibits invasion [37].

### 3.3. Chemo-Resistance

Chemo-resistance was studied in ovarian cancer. In a cohort of 287 high-grade ovarian serous cystadenocarcinoma patients from The Cancer Genome Atlas (TCGA) database, higher expression of NTR1 mRNA was significantly associated with platinum-resistant status (*p* = 0.0076) [25]. An NTR1 antagonist improved the responses to platinum salt therapy by inhibiting in vitro cell growth and survival. Using a monoclonal antibody directed against NT on ovarian human cancer cells inoculated in mice, Liu et al. showed that a reduction in NT/NTR1 activation improved the response to the platinum salt therapy as it decreased tumor size when cisplatin was combined with an NT antibody. It also facilitated nuclear platinum accumulation and reduced carboplatin efflux from the cells [25]. These results are consistent with Han et al.’s findings, making NT pathway inhibition a promising therapeutic associated with chemotherapy [57].

In summary, NT and its receptors are involved in critical oncologic processes, including proliferation, invasion, metastatic potential, and chemo-resistance. NT promotes tumor proliferation in breast and ovarian cancers. The NT/NTR1 axis plays a key role in both promoting proliferation and enhancing metastatic potential. Furthermore, NT is involved in chemo-resistance, particularly in ovarian cancer, linking NTR1 expression with platinum-resistant cases. This comprehensive understanding highlights the potential of NT and its receptors as therapeutic targets across multiple cancer types. The main data about the oncological processes associated with NT and its receptors are summarized in Table 2.

## 4. Clinical Features

### 4.1. Survival and Prognosis

Few studies account for survival data when it comes to NT expression and its receptors in hormone-sensitive cancers. 

To begin with, breast cancers, especially IDC expressing NTR1, were associated with a significantly worse prognosis than those with low receptor expression [19]. Moreover, high NTSR1 expression was linked to a larger tumor size, a grade 3 tumor, or a larger number of positive lymph nodes, making high NTSR1 expression an independent prognosis marker. NTR3 also seems to be linked to a worse prognosis, being associated with lymph node invasion in a cohort of 318 human breast cancer samples [37]. Consistently, high NTR3 expression seems to be associated with a poor prognosis for TN breast cancer, in particular for N+ cancer [36].

Concerning ovarian cancer, high NTR1 mRNA expression, in a cohort of 491 high-grade ovarian serous cystadenocarcinoma patients from TCGA database, was linked to a significantly worse prognosis with shorter progression-free survival. Surprisingly, NTR3 expression seemed to be correlated with a good prognosis [25]. 

Similarly, a high NTR1 mRNA expression level seemed to be linked to worse overall survival in endometrial cancer. Indeed, statistical analyses of 333 cases from TGCA cohort of uterine corpus endometrial carcinoma (UCEC) showed shorter overall survival and progression-free survival both in univariate and multivariate analysis independent from the histological grade, even when including only endometrioid carcinomas. These results were confirmed in a series of 100 consecutive cases of endometrial adenocarcinoma, showing that high immunohistochemical expression of NTR1 was significantly correlated with shorter overall survival (*p* < 0.001) [24]. 

### 4.2. Diagnosis and Pre-Therapeutic Strategies 

To improve tumor targeting when conventional imaging is insufficient, the neurotensinergic system has potential as a diagnostic tool, which is particularly studied in prostate and digestive cancers. NT receptors, especially NTR1, have been explored in positron emission tomography (PET) imaging. New nanoparticles labeled with radioisotopes, such as 64Cu-labeled NT analogs, were tested in PC3 (androgen-resistant prostate cancer cell line) cell xenografts and showed promise for identifying NTR-positive lesions with low uptake in normal tissues, which could help predict patient responses to NTR1-targeted therapies [58]. Further studies used ^68^Ga-labeled peptides functionalized on gold nanoparticles to enhance imaging of hormone-sensitive prostate and colon cancer cells in mice [59]. Similarly, ^18^F-DEG-VS-NT demonstrated strong tumor uptake in animal models, making NTR1 a promising target, particularly when prostate-specific membrane antigen (PSMA) expression is low [60]. Retrospective autoradiography studies also showed NTR1 positivity in PSMA-negative prostate lesions, reinforcing the potential of NTR1 imaging [61]. Other peptide-based imaging agents targeting NTR1 were successfully tested in xenografted mice with prostate cancer cells [62,63]. In pancreatic cancer, a radiopharmaceutical targeting neurotensin receptors has even been safely tested for the first time in three patients with ductal pancreatic adenocarcinoma [64].

NTR2 has recently emerged as a potential imaging target. A study on an ^18^F-labeled radioligand showed limited stability in vivo [65], but JMV-7488, a radiometalated NT analog targeting NTR2, demonstrated promising results in estrogen-positive breast cancer and prostate cancer animal models [33].

To address tumor heterogeneity, Ma et al. developed a heterodimer that targets both PSMA and NTR1, which showed prominent uptake in NTR1-positive/PSMA-negative and PSMA-positive/NTR1-negative prostate cancer xenografts [66]. This dual-targeting approach holds promise not only for imaging but also for therapeutic applications, as the heterodimer could be loaded with therapeutic agents.

Theranostics, which combines diagnosis, treatment, and follow-up, is an evolving field. The early success of radiolabeled somatostatin analogs in neuroendocrine tumors has spurred interest in NT-receptor-targeting agents for use in multiple cancers [58]. Peptide-based agents may serve as vehicles for delivering radioactivity to cancer cells, offering both diagnostic and therapeutic potential [67]. An exciting example of this approach is the use of Lutetium-177-labeled PSMA (Lu-PSMA), which has shown promise in treating metastatic prostate cancer by delivering targeted radiation to PSMA-expressing tumor cells. Similar strategies could target NT receptors, as demonstrated by Schulz et al., who found that a ^177^Lu-labeled NTR1-targeting agent effectively delayed tumor growth in NTR1-positive colon carcinoma xenografts, with significant reductions in tumor size [68].

### 4.3. Other Therapeutic Potentialities 

Several NTR agonists and antagonists have been developed for the study of NT binding and activity. Among them, SR48692 (Meclinertant), a selective NTR1 antagonist that has also demonstrated binding to NTR2 and SORT1 at higher concentrations, has been well studied. It was shown to reduce cell growth and apoptosis in different ovarian cancer cell lines [55] but also reduced the proliferation of LNCaP prostate cancer cells and prostate cell lines unresponsive to androgen deprivation therapy [69]. 

In animal models, daily administration of SR48692 reduced the volume and weight of TN breast cancer xenografts compared to control groups [21]. Additionally, SR48692 combined with carboplatin significantly decreased tumor growth in ovarian adenocarcinoma xenografts [25]. When combined with Enzalutamide, an androgen receptor inhibitor, SR48692 also reduced neuroendocrine differentiation and slowed tumor growth in prostate cancer xenografts, suggesting that targeting the NT system could delay castration-resistant prostate cancer progression [70]. SR48692 has also demonstrated radiosensitizing effects in PC3 xenografts, reducing tumor burden and enhancing radiation effects in vivo, independent of androgen receptor status [23]. 

NT-conjugated peptides have been developed to selectively deliver chemotherapeutics into tumor cells [71]. For example, PC3 cell viability was reduced by 70% when methotrexate was conjugated to NT, though free methotrexate had no effect. Similarly, NT-conjugated 5FdU became active in prostate cancer cells. New drug-conjugated NT peptides have been developed, showing cytotoxicity on human prostate cancer cells [72]. Additionally, NT-modified Camptothecin dimeric prodrug nanoparticles have shown enhanced tumor accumulation and reduced systemic toxicity in TN breast cancer models [73].

A peptide requiring SORT1 (TH1902) for internalization was recently tested and showed significant tumor regression in TN breast cancer murine xenografts when conjugated with docetaxel, without major adverse effects [36]. It also showed efficacy in ovarian cancer [36] and thus is being currently tested in a phase 1, open-label first-in-human study in solid cancer (ClinicalTrials.gov; Identifier: NCT04706962).

Chemical antagonists are not the only therapeutic options that may target the NT/NTR complex as new therapeutic drugs appear. Indeed, a monoclonal antibody (LF-NTS mAb) has recently been developed to target NTR1 in metastatic non-small-cell lung cancer and lower their aggressiveness. Adding an LF-NTS-targeted antibody to the standard of care (carboplatin/paclitaxel or cisplatin/pemetrexed) improved the treatment’s efficacy and showed toxicity in mice [74]. The same approach was used in ovarian cancer [25] and was recently studied in TN breast cancer [75]. 

Recent ligands for NT receptors are also addressed, such as SBI-553, a novel positive allosteric modulator of NTR1, whose detailed structural and action mechanism analyses have been studied by Krumm et al. [76]. A fluorescent molecular probe for the intracellular allosteric SBI-553 binding site of NTR1 was developed and employed in drug library screening, allowing for the identification of new chemotypes as binders for this pocket; as such, the development of intracellular allosteric ligands for NTR1 could be an interesting drug target [77].

In summary, most research on therapeutic options targeting NT and its receptors has not focused on female hormone-sensitive cancers. However, recent discoveries regarding the common oncologic processes involving the neurotensinergic pathway suggest that these receptors could become promising therapeutic targets in the future.

## 5. Conclusion and Perspectives

**Figure 1:**
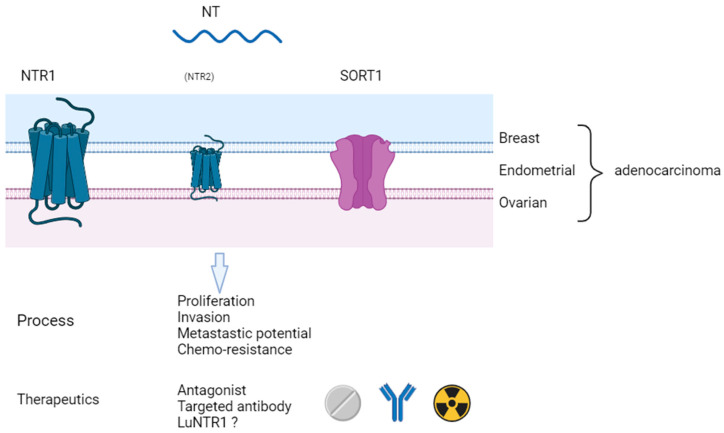
Synthesis of roles and perspectives for NT and its receptors in breast, ovarian, and endometrial cancers. NT: neurotensin; NTR1: neurotensin receptor 1; NTR2: neurotensin receptor 2; SORT1: sortilin.

The aim of this review was to synthesize current knowledge on the role of NT and its three receptors in breast, ovarian, and endometrial cancers—three hormone-sensitive cancers (Figure 1). While differences in NT receptor expression exist between these cancers, as well as between their hormone-sensitive and -insensitive cell lines, the role of NT and its receptors in oncologic processes, particularly in proliferation and invasion, appears consistent. Additionally, their impact on prognosis seems evident in most cases. Looking forward, the development of NT-receptor-targeted therapies, including theranostics and radioligand treatments, presents a promising avenue for personalized cancer treatment. These approaches, such as Lu-NTR1, exemplified by the success of agents like Lu-PSMA, could offer more precise and effective therapeutic options. However, further clinical studies are required to standardize these findings across different cancer types, so NT and its receptors can be fully harnessed as diagnostic and therapeutic tools in patient care.

## Figures and Tables

**Table 1:** Expression of NT and its receptors in female hormone-sensitive cancers.

Tissues	Type of Sample	NT	NTR1	NTR2	SORT1	References
**Breast**	Human breast adenocarcinoma	+/+++	+/+++	NA	+/+++	[19,21,36,37]
	Human normal breast tissue	−/+	−	NA	−	[19,21,37]
	MCF-7 (human ER-positive breast cancer CL)	NA	+++	+	+++	[21,32,33,37]
	MDA-MB-231 (human triple-negative breast cancer CL)	NA	+++	−	+++	[21,33,37]
**Ovary**	Human ovarian adenocarcinoma	+	+/+++	NA	+++	[25,35,38]
	Human normal ovarian tissue	+	−	NA	−	[25,35,38]
	SKOV3 (human epithelial ovarian cancer CL)	+	+++	−	+++	[25,35,38]
**Endometrium**	Human adenocarcinoma	+++	+/+++	NA	+/+++	[24,35]
	Human normal endometrium	−	−	NA	−	[24,35]
	HEC-1-A and HEC-1-B (human endometrial cancer CL)	NA	NA	NA	+	[35]

CL: cell line; ER: estrogen receptor; NA: not available; NT: neurotensin; NTR1: neurotensin receptor 1; NTR2: neurotensin receptor 2; SORT1: sortilin; −: no expression; +: weak expression; +++: strong expression.

**Table 2:** Oncological processes and key signaling pathways associated with NT and its receptors.

Processes	Tissues	Data	References
**Proliferation**	Breast cancer	JMV-449 increased DNA synthesis in MDA-MB-231 cells and inhibited apoptosis through Bcl-2 proto-oncogene activation in MCF-7 cells. NT overexpression increased tumor growth in MCF-7 xenografted mice. The NT/NTR1 complex showed a synergic effect with EGFR, HER2, and HER3. Silencing NTR1 in MDA-MB-231 xenografted mice reduced tumor volume.	[21,32,53,54]
	Ovarian cancer	Exogenous NT increased proliferation in the OCAR3 cell line. SORT1 silencing in Caov-4 cells increased apoptosis and decreased proliferation.	[39,55]
**Invasion and metastatic potential**	Breast cancer	While ER-positive NT-negative MCF-7 xenografts did not develop metastases, high-NT-expressing clones showed greater metastatic potential. The NT/NTR1 complex reduced basal cell adhesion and synergized with EGF. NTR1 activation led to MMP-9 expression. The knockdown of SORT1 in MDA-MB-231 cells inhibited invasion.	[21,37,53,54]
**Chemo-resistance**	Ovarian cancer	Higher expression of NTR1 mRNA was significantly associated with platinum-resistant status.	[54]

NT: neurotensin; NTR1: neurotensin receptor 1; EGFR: Epidermal Growth Factor Receptor; HER2: Human Epidermal Growth Factor Receptor-2; HER3: Human Epidermal Growth Factor Receptor-3; MMP-9: matrix metalloproteinase-9; SORT1: sortilin.
